# Assessment of Broadly Reactive Responses in Patients With MERS-CoV Infection and SARS-CoV-2 Vaccination

**DOI:** 10.1001/jamanetworkopen.2023.19222

**Published:** 2023-06-30

**Authors:** Hadeel T. Zedan, Maria K. Smatti, Swapna Thomas, Gheyath K. Nasrallah, Nahla M. Afifi, Ali Ait Hssain, Laith J. Abu Raddad, Peter V. Coyle, Jean-Charles Grivel, Muna A. Almaslamani, Asmaa A. Althani, Hadi M. Yassine

**Affiliations:** 1 Biomedical Research Center, Research Complex, Qatar University, Doha, Qatar; 2 Department of Biomedical Science, College of Health Sciences, Member of QU Health, Qatar University, Doha, Qatar; 3 Department of Biological and Environmental Sciences, College of Arts and Sciences, Qatar University, Doha, Qatar; 4 Qatar Biobank, Qatar Foundation, Doha, Qatar; 5 Medical Intensive Care Unit, Hamad Medical Corporation, Doha, Qatar; 6 Infectious Disease Epidemiology Group, Department of Population Health Sciences, Weill Cornell Medicine-Qatar, Doha, Qatar; 7 Virology laboratory, Hamad Medical Corporation, Doha, Qatar; 8 Deep Phenotyping Core, Sidra Medicine, Doha, Qatar; 9 Communicable Disease Center, Hamad Medical Corporation, Doha, Qatar

## Abstract

**Importance:**

In the ongoing COVID-19 pandemic, there remain unanswered questions regarding the nature and importance of the humoral immune response against other coronaviruses. Although coinfection of the Middle East respiratory syndrome coronavirus (MERS-CoV) with the SARS-CoV-2 has not been documented yet, several patients previously infected with MERS-CoV received the COVID-19 vaccine; data describing how preexisting MERS-CoV immunity may shape the response to SARS-CoV-2 following infection or vaccination are lacking.

**Objective:**

To characterize the cross-reactive and protective humoral responses in patients exposed to both MERS-CoV infection and SARS-CoV-2 vaccination.

**Design, Setting, and Participants:**

This cohort study involved a total of 18 sera samples collected from 14 patients with MERS-CoV infection before (n = 12) and after (n = 6) vaccination with 2 doses of COVID-19 mRNA vaccine (BNT162b2 or mRNA-1273). Of those patients, 4 had prevaccination and postvaccination samples. Antibody responses to SARS-CoV-2 and MERS-CoV were assessed as well as cross-reactive responses to other human coronaviruses.

**Main Outcomes and Measures:**

The main outcomes measured were binding antibody responses, neutralizing antibodies, and antibody-dependent cellular cytotoxicity (ADCC) activity. Binding antibodies targeting SARS-CoV-2 main antigens (spike [S], nucleocapsid, and receptor-binding domain) were detected using automated immunoassays. Cross-reactive antibodies with the S1 protein of SARS-CoV, MERS-CoV, and common human coronaviruses were analyzed using a bead-based assay. Neutralizing antibodies (NAbs) against MERS-CoV and SARS-CoV-2 as well as ADCC activity against SARS-CoV-2 were assessed.

**Results:**

A total of 18 samples were collected from 14 male patients with MERS-CoV infection (mean [SD] age, 43.8 [14.6] years). Median (IQR) duration between primary COVID-19 vaccination and sample collection was 146 (47-189) days. Prevaccination samples had high levels of anti-MERS S1 immunoglobin M (IgM) and IgG (reactivity index ranging from 0.80 to 54.7 for IgM and from 0.85 to 176.3 for IgG). Cross-reactive antibodies with SARS-CoV and SARS-CoV-2 were also detected in these samples. However, cross-reactivity against other coronaviruses was not detected by the microarray assay. Postvaccination samples showed significantly higher levels of total antibodies, IgG, and IgA targeting SARS-CoV-2 S protein compared with prevaccination samples (eg, mean total antibodies: 8955.0 AU/mL; 95% CI, −5025.0 to 22936.0 arbitrary units/mL; *P* = .002). In addition, significantly higher anti-SARS S1 IgG levels were detected following vaccination (mean reactivity index, 55.4; 95% CI, −9.1 to 120.0; *P* = .001), suggesting potential cross-reactivity with these coronaviruses. Also, anti-S NAbs were significantly boosted against SARS-CoV-2 (50.5% neutralization; 95% CI, 17.6% to 83.2% neutralization; *P* < .001) after vaccination. Furthermore, there was no significant increase in antibody-dependent cellular cytotoxicity against SARS-CoV-2 S protein postvaccination.

**Conclusions and Relevance:**

This cohort study found a significant boost in cross-reactive NAbs in some patients exposed to MERS-CoV and SARS-CoV-2 antigens. These findings suggest that isolation of broadly reactive antibodies from these patients may help guide the development of a pancoronavirus vaccine by targeting cross-reactive epitopes between distinct strains of human coronaviruses.

## Key Points

**Question:**

Is exposure to Middle East respiratory syndrome coronavirus (MERS-CoV) infection and SARS-CoV-2 vaccination associated with the generation of broadly reactive antibody responses?

**Findings:**

In this cohort study of 18 sera samples collected from 14 patients with MERS-CoV infection before and after COVID-19 vaccination, there were significantly boosted cross-reactive immune responses toward other human coronaviruses, with the strongest cross-recognition between SARS-CoV-2, SARS-CoV, and MERS-CoV.

**Meaning:**

These results suggest that COVID-19 vaccination might enhance preexisting immunity against MERS-CoV and SARS-CoV, and to a lesser extent, against other human coronaviruses.

## Teaser

This cohort study investigates the cross-reactive and protective humoral responses in patients exposed to Middle East respiratory syndrome (MERS-CoV) infection and SARS-CoV-2 vaccination.

## Introduction

In the past 2 decades, the world has faced 3 coronavirus outbreaks caused by the *Coronaviridae* family: severe acute respiratory syndrome (SARS) in 2002, Middle East respiratory syndrome (MERS) in 2013, and COVID-19 in 2019.^1^ The latter disease is caused by the currently circulating SARS-CoV-2, which is the seventh known human coronavirus (CoV) to naturally infect humans.^2^ Among this family, 4 seasonal CoVs—229E, NL63, OC43, and HKU1—cause mild respiratory infections and account for approximately 5% to 30% of common cold infections.^3^ However, SARS-CoV, MERS-CoV, and SARS-CoV-2 cause severe acute respiratory illnesses and even death, with fatality rates up to 10%, 37%, and 3.7%, respectively.^1,4^

A growing number of studies have suggested potential cross-reactivity between SARS-CoV-2 and other human CoVs due to the high sequence homology, leading to cross-reactive immune responses.^5^ Phylogenetic analysis revealed 78% and 50% sequence homology of SARS-CoV-2 to SARS-CoV and MERS-CoV, respectively.^4^ Therefore, due to this pronounced similarity, cross-reactive antibodies targeting cognate antigenic epitopes are expected. Additionally, reactivity to SARS-CoV-2 was detected in prepandemic samples of seropositive individuals for other human CoVs.^6^ Ideally, broadly neutralizing antibodies (NAbs) targeting different CoVs could be elicited,^7^ which would guide the development of a pan-CoV vaccine.

As of February 2022, a total of 2585 laboratory-confirmed MERS cases and 890 related deaths were reported worldwide,^8^ of which approximately 2184 cases (84.5%) were in the Kingdom of Saudi Arabia. In Qatar, 30 human cases were reported since the MERS-CoV outbreak started in 2012 and through 2022.^9^ Although coinfection with SARS-CoV-2 has not been documented yet, many people previously infected with MERS received the COVID-19 vaccine. However, there is limited data on how preexisting MERS immunity may shape the response to SARS-CoV-2 following infection or vaccination. Here, we characterized cross-reactive antibody responses in patients exposed to MERS-CoV and SARS-CoV-2 through infection and vaccination, respectively. We also assessed protective immune responses in these patients, including NAbs and antibody-dependent cellular cytotoxicity (ADCC).

## Methods

### Study Design, Ethical Approval, and Sample Collection

This cohort study was approved by the institutional review board at Qatar Biobank, Hamad Medical Corporation, and Qatar University. Written informed consent was obtained for all participants before study enrollment. The study followed the Strengthening the Reporting of Observational Studies in Epidemiology (STROBE) reporting guideline for cohort studies.^10^ A total of 14 patients enrolled in this study who had a history of previous MERS-CoV infection and had sera samples collected either before (n = 12) or after (n = 6) receiving 2 doses of COVID-19 mRNA vaccines (BNT162b2 [Pfizer-BioNTech] or mRNA-1273 [Moderna]). Among these patients, we were able to obtain follow-up samples from 4 patients.

For the control group, sera samples collected from patients who had recovered from COVID-19 (n = 10) and naive-vaccinated individuals with COVID-19 mRNA vaccine (n = 10) who had no history of SARS-CoV-2 infection (confirmed by Architect anti-N immunoglobin G [IgG] immunoassay). The selected samples for the control group were collected between 1 and 6 months following infection with SARS-CoV-2 or vaccination with COVID-19 mRNA vaccine.

### Serology Testing for Anti–SARS-CoV-2 Antibodies

All sera samples were tested using CL-900i automated analyzer (Mindray Bio-Medical Electronics),^11^ which detects antibodies targeting SARS-CoV-2 antigens including (1) anti-spike (S) and N IgG, (2) anti-S and N immunoglobin M (IgM), (3) anti-S–receptor-binding domain (RBD) IgG, (4) anti-S-RBD total antibodies (IgG, IgA, and IgM), and (5) NTAb assay. Anti-SARS-CoV-2 S1 IgA was measured using the Euroimmun anti–SARS-CoV-2 IgA enzyme-linked immunosorbent assay (ELISA).^12^ All tests were performed according to the manufacturer’s instructions.

### Microarray Immunoassay for Assessing Cross-Reactivity With Human CoVs

A bead array was used to detect cross-reactive antibodies with other human CoVs. The assay consists of 11 carboxymethylated beads with 11 different intensities of UV-excitable dye (Spherotech). Each bead-set was individually coupled to histidine-tagged recombinant human CoV proteins expressed in human cells (Acro Biosystems). Five SARS-CoV-2 proteins and/or protein fragments were included: S trimer, RBD, S1, nucleocapsid (N), and envelope protein. Also, the S1 subunit of all other human CoVs were included: SARS-CoV-S1, MERS-CoV-S1, 229E-S1, NL63-S1, HKU1-S1, and OC43-S1. Coupling was performed according to a previously published procedure^13^ with a slight modification consisting of buffer exchange of reconstituted lyophilized recombinant proteins with phosphate-buffered saline (PBS) pH 7.4 using Pierce Zeba columns.

#### Microarray Serological Assay

Serum samples diluted 1:20 in assay buffer (10mM Tris-HCl, pH 7.5, 0.1% BSA, 0.01% polyethylene glycol sorbitan monolaurate [Tween 20]) were incubated with the bead array (2000 microspheres for each peak) in a total volume of 50 μL in a multiscreen HV filter plate (Millipore MSHVN4510) under shaking at 800 rpm for 35 minutes at room temperature. After 3 vacuum washes with assay buffer, the microspheres were incubated with 50 μL assay buffer containing 0.6 μg/mL of AlexaFluor 488-labeled goat antihuman IgG polyclonal antibodies (SouthernBiotech 2040-30), 0.63 μg/mL PE-labeled goat antihuman IgA polyclonal antibodies (Jackson ImmunoResearch 109-115-011), and 1.2 μg/mL AlexaFluor 647-labeled goat antihuman IgM polyclonal antibodies (SouthernBiotech 2020-31) for 20 minutes at room temperature under agitation at 800 rpm. The microspheres were then vacuum washed 3 times in wash buffer (10mM Tris-HCl; pH 7.5; 0.05% Tween 20), resuspended, acquired, and analyzed on a BD FACS Symphony A5 equipped with UV (355 nm), violet (450 nm), blue (488 nm), yellow-green (561 nm), and red (633 nm) lasers and a high-throughput sampler.

#### Data Acquisition and Analysis

Bead classification was performed by defining 11 gates in bivariate plots of UV 515 (UV excitation, emission 515/30) fluorescence vs violet 525 (405 excitation, emission 525/50) fluorescence. Each gated bead population was analyzed for the presence of IgM, IgG, and IgA, revealed by the fluorescence intensity in B-520 for AlexaFluor488 (488 nm excitation, 525/50 emission), YG-586 PE (561 nm excitation, 586/14 emission), R670 for AlexaFluor647 (640 excitation, 670/30 emission). The data were analyzed using FlowJo software version 10 (BD Biosciences). A mean of 300 beads per region was acquired. The median fluorescence intensity (MFI) of each bead peak in the aforementioned fluorescent channels was used to calculate the positivity index for each sample (ratio of participant MFI/pooled negative control MFI for the same antigen/isotype). Pooled negative controls were collected prior to 2018.

### Neutralization Assays

#### Pseudovirus Production, Titration, and Neutralization Test

A pseudovirus neutralization test (PVNT) was used to measure NAbs against SARS-CoV-2 and MERS-CoV in vitro*.* The pseudovirus expresses the S glycoprotein, which mediates entry into the host cells by binding to the human receptor. Pseudoviruses expressing wild-type SARS-CoV-2 (Wuhan-Hu-1) and MERS-CoV S were prepared using human embryonic kidney (HEK293T) cells (ATCC) infected with vesicular stomatitis virus ΔG-luc seed virus as previously described.^14^ For the pseudovirus titration assay, HEK293T cells were used, as described by Wang et al.^15^ Cells and plasmids were kindly provided by Viral Pathogenesis Laboratory, Vaccine Research Center, National Institutes of ‎Health (NIH). Briefly, HEK293T cells were plated at 1 × 10^4^ cells per well in a 96-well white/black isoplate (PerkinElmer) and cultured overnight. The following day, the culture medium was removed, and 2-fold serial dilutions of the pseudovirus were added to the cells and incubated for 2 hours. Then, 100 μL of fresh medium was added; and after 72 hours, cells were lysed using 1 × lysis buffer, and 50 μL of luciferase substrate (Promega Bio-Glo Luciferase Assay System) was added to each well. Luciferase activity was measured using a luminescence plate reader (Tecan Infinite 200 PRO). Following pseudovirus titration, the neutralization assay was carried out as described elsewhere.^15^ Luciferase activity was measured using a luminescence plate reader (Tecan), and percentage inhibition was calculated for each sample.

#### Surrogate Virus Neutralization Test

A surrogate virus neutralization test was used to detect NAbs that block the interaction between SARS-CoV-2 RBD and human angiotensin-converting enzyme 2 (ACE2) receptors.^16^ The assay is an ELISA-based inhibition test developed by GenScript (GenScript Biotech)^17,18,19^ to serologically screen for NAbs targeting SARS-CoV-2 RBD. The assay was carried out according to manufacturer’s instructions. Result interpretation was as follows: percentage inhibition greater than or equal to 30% was considered positive (detectable NAbs), and less than 30% was considered negative (nondetectable NAbs).

### ADCC Assay

ADCC activity was assessed using a commercial ADCC reporter bioassay kit (Promega) containing ready-to-use ADCC effector cells (Jurkat-FcγRIIIa-NFAT-Luc, V158 high-affinity variant).^20^ This assay is an Fc Effector Reporter Bioassay that measures the ability of serum antibodies to activate the nuclear factor of activated T cells (NFAT) pathway through FcγRIII (the pathway that initiates ADCC in NK cells) in the presence of target antigens coated on a 96-well plate. The assay replaces traditional primary cell–based assays with Jurkat cells that stably express human FcγRIIIa V158 and NFAT-induced luciferase. The ADCC reporter assay was performed in accordance with the manufacturer’s instructions. Briefly, 96-well plates were coated overnight at 4 °C with purified SARS-CoV-2 S (SinoBiological, catalog number 40589-V08H4) and N (SinoBiological, catalog number 40588-V08B) proteins (300 ng/well and 100 ng/well, respectively) in 1 × PBS. Wells were washed with PBS/0.05% Tween 20 and blocked with 5% BSA. Then, serially diluted sera were added and incubated at 37 °C for 2 hours. ADCC effector cells were then added to each well (75 000 cells/well), and incubated overnight at 37 °C. After incubation, a luciferase reagent was used to measure luminescence activity using a luminescence plate reader. ADCC activity was reported as fold change in relative light units (RLUs) which was calculated as follows: fold of induction = RLU of induced / RLU of no serum control.

### Statistical Analysis

Statistical analysis was performed using GraphPad Prism version 9.0 software and Microsoft Excel 2013 from February 2022 to March 2023. The difference between groups was evaluated using 1-way analysis of variance. For all analyses, 2-sided *P* < .05 was considered statistically significant.

## Results

We characterized a total of 18 samples collected from 14 patients (all male; mean [SD] age, 43.8 [14.6] years) who had MERS-CoV infection; 12 samples were taken before COVID-19 vaccination, and 6 samples were taken after COVID-19 vaccination. Among those patients, 4 had follow-up samples. The median (IQR) duration between second dose administration and sample collection was 146 (47-189) days. Patients’ demographic and clinical data are shown in the eTable in Supplement 1. We first performed serologic characterization for anti–SARS-CoV-2 immune responses using 6 different automated immunoassays, including IgM, IgG, IgA, NAbs, and total antibodies targeting different antigens (eFigure 1 in Supplement 1). Cross-reactivity with other human CoVs was assessed using a microarray immunoassay targeting main antigens of SARS-CoV-2 and the S1 protein of SARS-CoV, MERS-CoV, and the 4 common human CoVs. Functional antibodies were also assessed, including NAbs (against MERS-CoV and SARS-CoV-2) and ADCC activity (against SARS-CoV-2 S and N proteins).

### Anti–SARS-CoV-2 Antibody Responses

**Figure 1.**
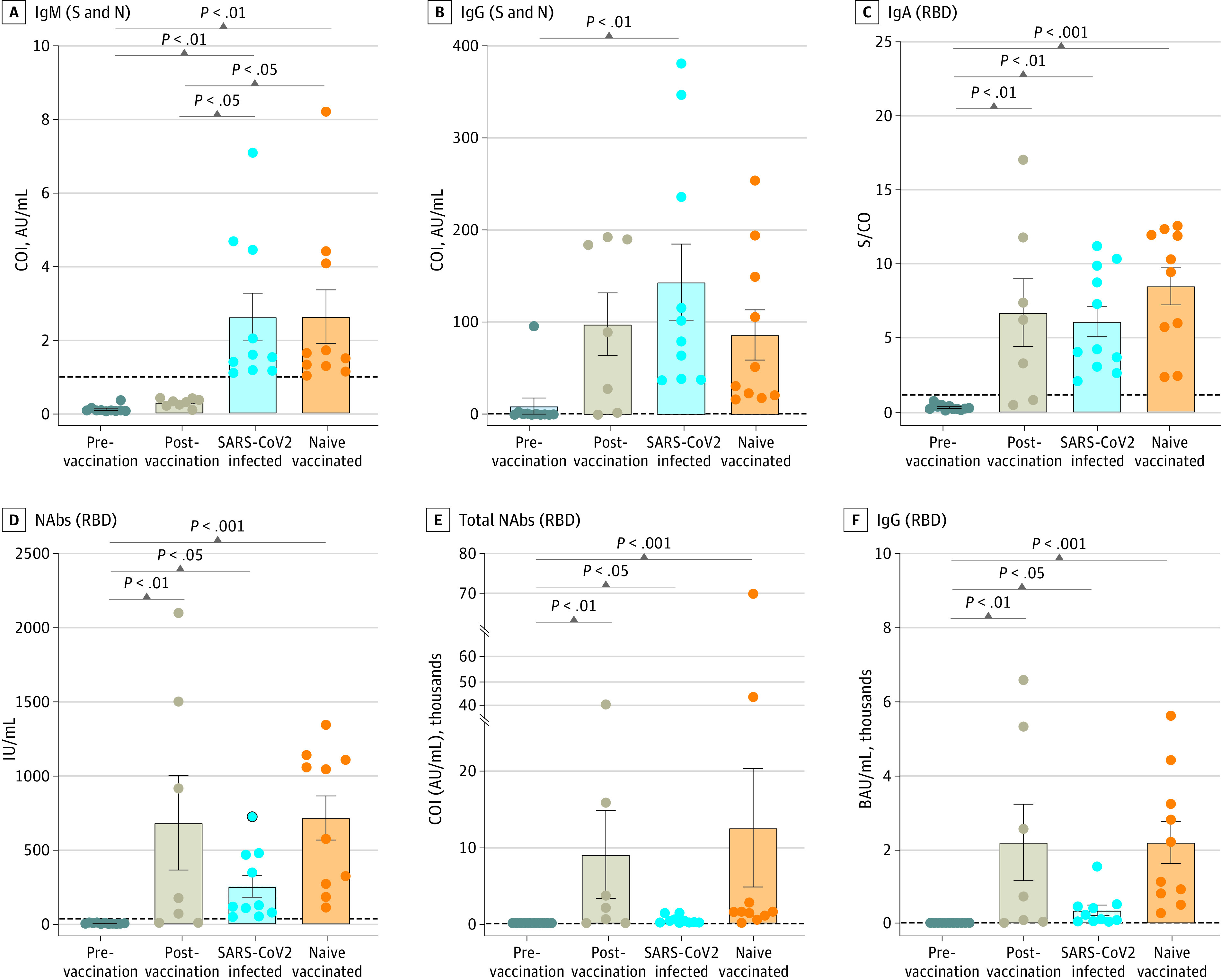
Antibody Responses Against SARS-CoV-2 in Samples Collected From Patients With Middle East Respiratory Syndrome Coronavirus (MERS-CoV) Before and After COVID-19 Vaccination Figure shows dot plot of antibody responses against SARS-CoV-2 in samples collected from patients with MERS-CoV before and after COVID-19 vaccination. Patients who had SARS-CoV-2 infection and individuals with COVID-19 vaccination (with no history of previous infections) served as controls. A, immunoglobin M (IgM) antibodies against SARS-CoV-2 spike (S) and nucleocapsid (N) proteins. B, IgG antibodies against S and N proteins. C, IgA antibodies against the receptor-binding domain (RBD). D, Neutralizing antibodies (NAbs) against the RBD. E, Total antibodies against the RBD. F, IgG antibodies against the RBD. *P* values were calculated using a 1-way analysis of variance test. AU indicates arbitrary units; BAU, binding antibody units; COI, cutoff index; S/CO, signal/cutoff.

An automated immunoassay was used to measure anti–SARS-CoV-2 antibodies (IgM, IgG, NAbs, and total antibodies). IgM and IgG responses against combined S and N antigens showed no significant difference postvaccination (Figure 1A and 1B). However, significantly higher levels of mean anti-RBD total antibodies (8955.0 arbitrary units (AU)/mL; 95% CI, −5025.0 to 22936.0 AU/mL; *P* = .002), IgA (6.7 signal/cutoff [S/CO]; 95% CI, 1.1 to 12.3 S/CO; *P* = .006), IgG (2182.0 binding antibody units [BAU]/mL; 95% CI, −359.4 to 4722.0 BAU/mL; *P* = .005), and NAbs (683.8 IU/mL; 95% CI, −97.73 to 1465.0 IU/mL; *P* = .008) were seen postvaccination (Figure 1C-1F). IgG and IgA levels postvaccination were comparable with those of patients with SARS-CoV-2 infection and naive (not infected)-vaccinated individuals (Figure 1B and 1C), whereas NAbs, total, and IgG (anti-RBD) were comparable with those of naive-vaccinated participants (Figure 1D-1F). Among the 4 participants who had follow-up samples, 3 showed higher postvaccination antibody responses, especially anti-RBD NAbs (4990.0 IU/mL; 95% CI, 112.9 to 9867.0 IU/mL; *P* = .02) and IgG (4162.0 BAU/mL; 95% CI, −1192.0 to 9517.0 BAU/mL; *P* = .04) (eFigure 2C and 2E in Supplement 1). However, 1 patient showed a limited response.

### Cross-Reactivity With Human CoVs

**Figure 2.**
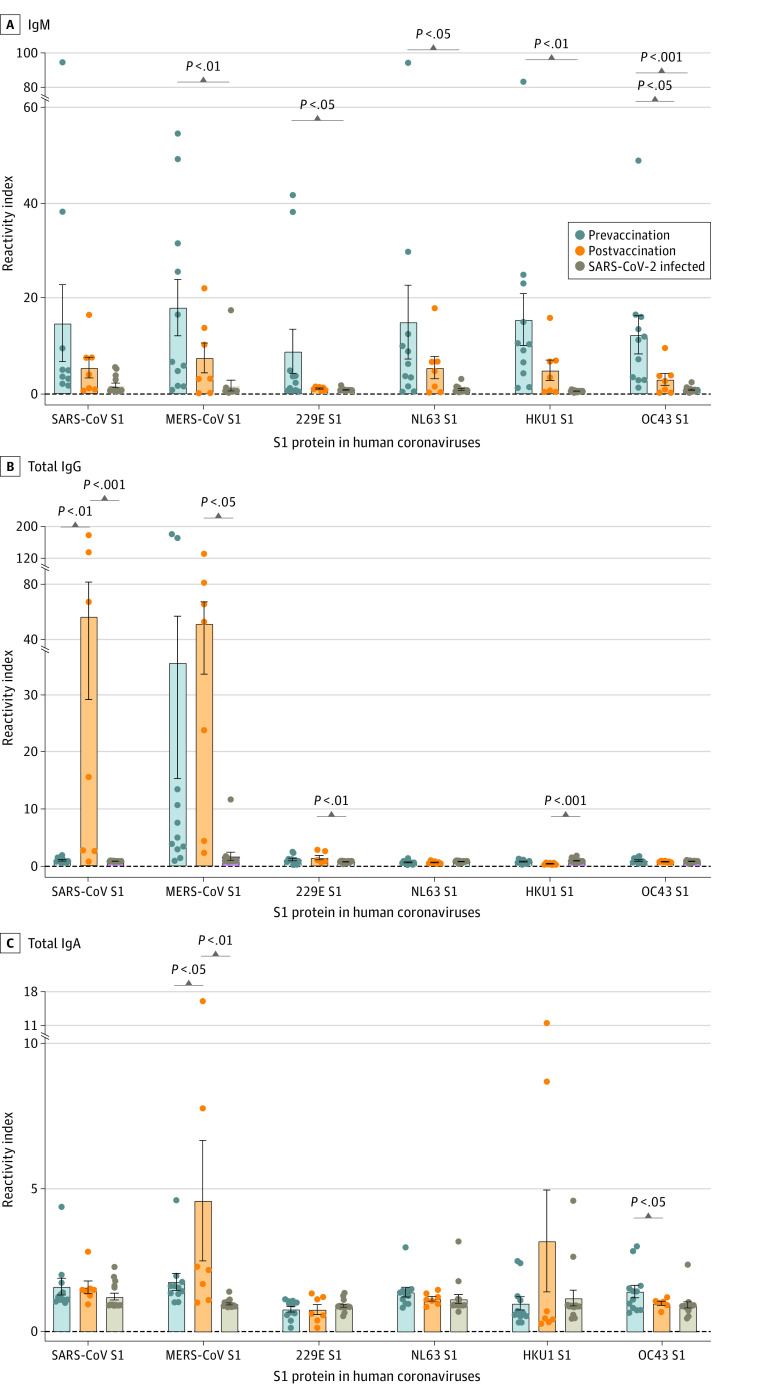
Antibody Responses to the Spike Protein S1 Subunit of Other Human Coronaviruses in Prevaccination and Postvaccination Samples Compared With Patients Who Had SARS-CoV-2 Infection Figure shows immunoglobin M (IgM) antibody responses (A), total IgG antibody responses (B), and total IgA antibody responses (C). *P* values were calculated using *t* test. MERS-CoV indicates Middle East respiratory syndrome coronavirus.

To assess cross-reactivity, all samples were screened using a microarray immunoassay against the S1 antigen of all human CoVs (Figure 2) as well as 5 SARS-CoV-2 antigens (eFigure 3 in Supplement 1). Samples collected from patients with SARS-CoV-2 infection were used as a control group. As shown in Figure 2A, prevaccination samples showed significantly higher levels of anti-MERS S1 IgM with reactivity index ranging between 0.80 and 54.7 (mean reactivity index, 18.1; 95% CI, 4.9 to 31.3; *P* = .001). These samples also showed significantly higher levels of anti-S1 IgM against seasonal CoVs 229E (8.9; 95% CI, −1.5 to 19.3; *P* = .04), NL63 (15.0; 95% CI, −2.3 to 32.3; *P* = .03), HKU1 (15.6; 95% CI, 3.5 to 27.7; *P* = .003), and OC43 (12.4; 95% CI, 3.4 to 21.4; *P* < .001) compared with patients with SARS-CoV-2 infection. No significant difference in IgM in prevaccination compared with postvaccination samples was seen across all tested antigens, except for OC43 (12.4; 95% CI, 3.4 to 21.4; *P* = .03). In addition, higher total IgG levels were seen postvaccination against SARS-CoV S1 (55.4; 95% CI, −9.1 to 120.0; *P* = .001) than prevaccination. These samples also showed higher total IgG levels against SARS-CoV S1 (55.4; 95% CI, −9.1 to 120.0; *P* = .004), MERS-CoV S1 (50.0; 95% CI, 9.5 to 90.5; *P* = .003) and 229E S1 (1.4; 95% CI, 0.5 to 2.3; *P* = .009) compared with patients with SARS-CoV-2 infection. Conversely, anti–HKU1-S1 total IgG was lower postvaccination (0.3; 95% CI, 0.2 to 0.5; *P* < .001), noting that the overall response against this virus was very low. Total IgA showed lower responses than IgM and IgG but was significantly higher against MERS-CoV S1 postvaccination compared with prevaccination (4.6; 95% CI, −0.6 to 9.8; *P* = .049) and the control group (4.6; 95% CI, −0.6 to 9.8; *P* = .005).

There were significantly higher IgG levels postvaccination against SARS-CoV-2 S trimer (mean reactivity index, 5.6; 95% CI, 1.4-9.9; *P* < .001), S1 (40.9; 95% CI, 6.2-75.7; *P* < .001), and RBD (20.2; 95% CI, 0.8-39.7; *P* < .001). Similarly, higher total IgA levels against SARS-CoV-2 S1 (2.8; 95% CI, 0.8-4.8; *P* = .007) and RBD (1.8; 95% CI, 1.1-2.4; *P* = .02) were detected postvaccination compared with prevaccination. Notably, prevaccination samples showed high levels of anti–SARS-CoV-2 IgM (eFigure 3A in Supplement 1) and IgA (eFigure 3C in Supplement 1).

We also examined the magnitude of IgG subclasses and there was no significant difference in IgG1 (induced in response to soluble protein antigens and membrane proteins^21^) across all tested antigens (eFigure 4A in Supplement 1). IgG2 (plays a role in protection against protein antigens but mainly responsible for anticarbohydrate IgG responses against bacterial capsular polysaccharide antigens^21^) against S1 of SARS-CoV (mean reactivity index, 3.9; 95% CI, 0.3 to 7.5; *P* = .01), and to a lesser extent against MERS-CoV, was higher postvaccination (eFigure 4B in Supplement 1). IgG3 (potent proinflammatory antibody and effective in the induction of effector functions^21^) targeting SARS-CoV S1 (1.2; 95% CI, 1.0 to 1.3; *P* = .003) were also higher postvaccination (eFigure 4C in Supplement 1). As for IgG4 (formed following repeated or long-term exposure to antigens in a noninfectious setting such as allergies^21^), there was no difference across all antigens except for SARS-CoV S1 (1.3; 95% CI, 0.9 to 1.7; *P* = .02), which was higher postvaccination (eFigure 4D in Supplement 1).

IgA subclasses (IgA1 and IgA2) were also assessed across all antigens, but there was no significant difference in both subclasses postvaccination (eFigure 5 in Supplement 1). Still, there was a noticeable increase in IgA1 against MERS- and HKU1-S1, as well as IgA2 against HKU1-S1 in at least 1 patient.

When separately analyzing the follow-up samples, 3 patients showed significantly higher levels of cross-reactive antibodies after vaccination to different CoVs, especially to MERS-CoV (IgM, total IgG, and IgG1) and SARS-CoV (IgM, total IgG, IgG1, and IgG2). Notably, patients 3 and 4 also showed higher anti-S1 IgM levels against human CoV-NL63, CoV-HKU1, and CoV-OC43, whereas patient 1 showed higher IgG1 and IgG3 levels against 229E-S1 (eFigure 6 in Supplement 1).

**Figure 3.**
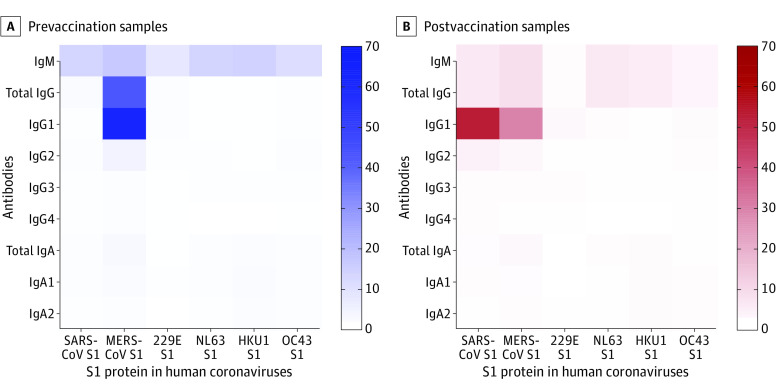
Heatmap Analysis of Antibodies Against the Spike Protein S1 Subunit of Other Human Coronaviruses Measured Using Microarray Immunoassay in Prevaccination Heatmap analysis of antibodies against the S1 protein of other human coronaviruses measured using microarray immunoassay in prevaccination (A) and postvaccination (B) samples. Reactivity indices are shown in light blue or light red for low values and dark blue or dark red for high values. IgA indicates immunoglobin A; IgG, immunoglobin G; IgM, immunoglobin M; MERS-CoV, Middle East respiratory syndrome coronavirus.

The reactivity indices of each antibody against the 11 antigens were used to construct a heatmap (Figure 3). The heatmap’s color scale, ranging from light to dark blue or red, corresponds with low and high reactivity indices. The results showed different levels of cross-reactivity in prevaccination and postvaccination sera with different human CoVs in different patients. Prevaccination sera showed high anti–MERS-CoV-S1 total IgG. These samples also showed some cross-reactivity with total IgG and IgG1 against SARS-CoV-2 N and S1. IgM and IgA against seasonal human CoVs, 229E, HKU1, and OC43, also showed some cross-reactivity in prevaccination samples (Figure 3A). Postvaccination samples showed very high reactivity for IgG1 against SARS-CoV-2 N, S1, and RBD. Furthermore, these samples showed significant cross-reactivity anti–SARS-CoV and anti–MERS-CoV S1 (Figure 3B).

### Neutralizing Antibody Responses

**Figure 4.**
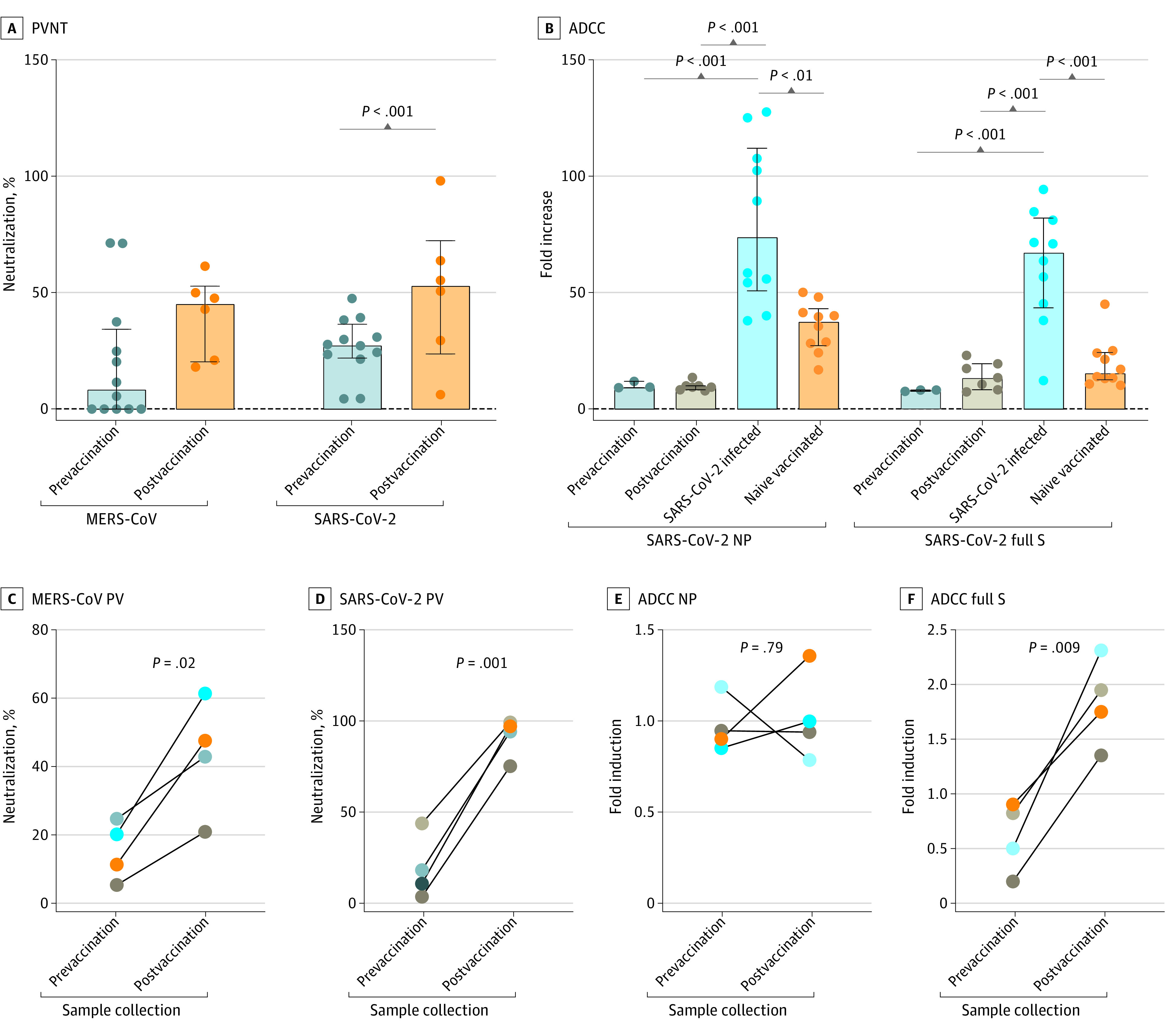
Assessment of Neutralizing and Antibody-Dependent Cellular Cytotoxicity (ADCC) Activity Against SARS-COV-2 and MERS-CoV A, Neutralization of pseudoviruses expressing MERS-CoV and SARS-CoV-2 spike (S) protein in prevaccination and postvaccination samples. B, ADCC activity against SARS-CoV-2 nucleocapsid protein (NP) and full S. The figure also compares the assessed groups with controls of patients with SARS-CoV infection and COVID-19–vaccinated individuals with mRNA vaccines. C and D, Neutralization of pseudoviruses expressing MERS-CoV (C) and SARS-CoV-2 (D) S protein in patients with follow-up samples collected before and after COVID-19 vaccination. E and F, ADCC activity against SARS-CoV-2 NP (E) and full S (F) in patients with follow-up samples collected before and after COVID-19 vaccination. *P* values were calculated using paired *t* test or 1-way analysis of variance test. PV indicates pseudovirus; PVNT, pseudovirus neutralization test.

Neutralization of pseudoviruses expressing MERS-CoV and SARS-CoV-2 S proteins was also assessed. Overall, there was no significant increase in MERS-CoV neutralization postvaccination, whereas SARS-CoV-2 neutralizing activity was significantly increased (50.5% neutralization; 95% CI, 17.6%-83.2% neutralization; *P* < .001) (Figure 4A). However, when assessing only those with follow-up samples, all 4 patients responded to COVID-19 vaccination, with significantly higher neutralization against both MERS-CoV (43.2; 95% CI, 16.6-69.8; *P* = .02) and SARS-CoV-2 pseudoviruses (91.3; 95% CI, 173.9-108.6; *P* = .001) (Figure 4C and 4D).

### ADCC Activity

ADCC activity was measured to assess anti-S and anti-N antibodies with Fc effector function activity. Overall, no significant difference was shown against both N and S proteins after vaccination (Figure 4B). However, sera collected from patients who had SARS-CoV-2 infection showed higher ADCC levels against the N protein (median [IQR] fold induction, 7.4-fold [5.1-fold to 11.2-fold]; *P* < .001) and the S protein (6.7-fold [4.3-fold to 8.2-fold]; *P* < .001) compered with prevaccination and postvaccination samples. Furthermore, sera collected form naive-vaccinated participants showed similar levels of ADCC activity against the N protein (median [IQR] fold induction, 3.8-fold [2.7-fold to 4.3-fold]) and the S protein (1.3-fold [1.3-fold to 2.4-fold]) compared with prevaccination and postvaccination samples. Of those with follow-up samples, all 4 patients showed significantly higher ADCC activity against SARS-CoV-2 S protein (Figure 4F), albeit not significant compared with SARS-CoV-2 infected participants, whereas no difference was seen against the N protein (Figure 4E).

## Discussion

We investigated the overall antibody responses in individuals exposed to MERS-CoV infection and SARS-CoV-2 vaccination. The overall changes in antibody subclass, isotype, neutralization, and Fc-mediated effector functions were assessed. Although we started with 12 patients, postvaccination samples were only collected from 6 individuals, 2 of whom did not have a prevaccination sample. Hence, all initial analyses were done to compare prevaccination (n = 12) to postvaccination (n = 6) sera, followed by a detailed analysis of those patients with follow-up samples (n = 4). Still, patients demonstrated different profiles of binding and neutralizing activities, and we identified 4 patients who elicited broadly reactive responses to multiple betacoronaviruses, including SARS-CoV, MERS-CoV, and SARS-CoV-2.

Exposure to MERS-CoV followed by SARS-CoV-2 antigens elicited high antibody responses, including total Ig, IgA, IgG, and NAbs, against SARS-CoV-2 S protein and RBD. First, we tested the antibody response to combined S and N antigens in 1 assay, which showed no boost in IgM levels after vaccination. In the context of COVID-19, vaccine-induced IgM responses are considered less characterized in terms of timing and the role of preexisting immunity. However, a recent study discussed the crucial role of IgM in developing protective immunity following vaccination with the BNT162b2 vaccine in individuals with and without SARS-CoV-2 infection.^22^ The coordinated anti-S IgM and IgG responses were associated with higher virus-neutralizing activity and increased protective immunity compared with IgG alone. Also, the absence of or minimal anti-S IgM following vaccination was suggested to result from preexisting immunity to cross-reactive human CoVs. Similarly, declines in anti-S IgM titers, along with rapid decay of NAbs, were reported in recovered COVID-19 patients.^23^ These observations suggest that at least part of the neutralizing activity is mediated by anti-S IgM and could possibly explain the apparent paradox between the stable IgG levels and declining neutralization capacity following SARS-CoV-2 infection and vaccination.

No statistically significant boost in IgG response against combined S and N antigens was observed after vaccination, noting that 2 individuals did not elicit a detectable response. However, significantly higher IgG responses were observed in patients with follow-up samples. The low overall response could be due to several factors including the time of sample collection, particularly that some samples were collected within 4 to 6 months postvaccination at which the immune response may have declined. However, recent studies have shown that mRNA vaccine-induced immunity produce high NAb titers which rapidly declines within 6 to 8 months postvaccination. Therefore, these patients are still likely to have detectable antibody responses. Another reason could be because the assay used to measure these antibodies targets SARS-CoV-2 S and N proteins and these patients received an mRNA vaccine, which encodes only the S protein.^24^ However, using the pVNT and automated CLIA assay, high titers of NAbs, predominantly anti-S and anti-RBD IgG, were detected postvaccination. Similar high titers of IgA, IgG, and total Ig against the RBD were also obtained. IgG2 and IgG3 against SARS-CoV-2 S1 were significantly increased postvaccination but not against the S trimer, suggesting that these antibodies do not target the S2 subunit, but mainly S1 and the RBD.^25^

Given the relatively high seroprevalence of common CoVs in the population, there have been questions related to the potential influence of preexisting immunity on the overall trajectory of the humoral immune response to other CoVs, including SARS-CoV-2. Therefore, we profiled anti-S1 antibody response across all human CoVs and found significant cross-reactivity between these betacorovaviruses, particularly SARS-CoV and SARS-CoV-2. Cross-reactive IgG, predominantly IgG1, IgG2, and IgG3, were induced against SARS-CoV after vaccination which was consistent with the levels of anti–SARS-CoV-2 antibodies. Given that these 2 CoVs share a substantial identity (approximately 78%) and potentially share antigenic epitopes capable of inducing adaptive immune responses, the generation of cross-reactive antibodies is not surprising.^3^ Similar corroborating evidence has been reported in several studies which showed significant cross-reactivity between SARS-CoV-2 and SARS-CoV, targeting predominantly non-RBD regions and, to a lesser extent, the RBD domain.^26^ However, weak cross-reactive NAbs to both CoVs were also reported, suggesting that a subset of these cross-reactive binding epitopes might be bona fide neutralizing epitopes.

Unlike SARS-CoV, the common human CoVs share limited sequence identity with SARS-CoV-2 S protein ranging between 23% to 25% for α-CoVs (229E and NL63) and 29% for β-CoVs (HKU1 and OC43).^27^ Thus, a little potential cross-reactivity between these CoVs would be expected. Indeed, cross-reactive IgG to seasonal human CoVs was only detected in a few patients, where 1 patient showed higher anti-229E IgG levels despite having low anti–SARS-CoV-2 antibodies. Additionally, 2 individuals showed slightly higher IgA antibody responses to HKU1 S1 antigen postvaccination. This could indicate that previous exposure to similar CoV epitopes may elicit cross-reactive responses to other human CoVs, despite sharing low sequence homology. Previous studies found that SARS-CoV-2 infection or vaccination elicits cross-reactive antibodies that target SARS-CoV-2 and related human CoVs.^28^ Also, this might be due to subsequent infection with seasonal human CoVs since they are widely circulating among populations and infections with these CoVs happen frequently.^29^

Additionally, higher levels of serum anti-S-protein antibodies against endemic human CoV were found in convalescent COVID-19 donors. These antibodies were linked to preexisting S-protein-specific cross-reactive memory B cells to human CoVs that might have been activated after SARS-CoV-2 infection.^30^ Also, infection with endemic human CoVs was shown to produce little cross-reactivity to SARS-CoV and MERS-CoV where patients with SARS were reported to have increased levels of anti–HCoV-229E, anti–HCoV-NL63, and anti–HCoV-OC43 antibodies in paired acute/convalescent samples.^31^

It is worth mentioning that to assess cross-reactivity, we used the S1 antigen in the microarray immunoassay. Typically, conservation between the different CoV S antigens is observed in the S2 subunit, not S1, and hence, the target for cross-reactive antibodies.^32^ Other studies also reported that cross-reactive monoclonal antibodies primarily target the more conserved S2 subunit, which was shown to display activities against a broader range of human CoVs.^30^ Therefore, further analysis using whole S protein along with a neutralization assay may reveal a different outcome.

Interestingly, MERS-CoV–seropositive samples collected before vaccination showed higher IgM cross-reactivity with the S1 antigen of all 7 human CoVs compared with postvaccination. However, when assessing those with follow-up samples, 3 patients showed increased levels of anti-MERS-CoV IgM antibodies, and 2 patients showed increased anti-SARS-CoV and anti-seasonal human CoVs (NL63, HKU1, and OC43) IgM postvaccination. This might be related to low assay specificity due to the polymeric nature of IgM, as cross-reactive anti-SARS-CoV-2 IgM antibodies were also detected in pre-COVID-19 samples.^33^ Furthermore, these prevaccination IgM responses could be related to the cross-reactivity induced against other human CoVs following infection due to isotype class-switching.

Notably, our study showed a significant increase in NAbs against MERS-CoV following vaccination. Cross-reactive NAbs against MERS-CoV were also detected at low titers in convalescent SARS sera in China.^34^ In that study, 60% of SARS sera had detectable antibody titers to MERS-CoV, and 25% had anti–MERS-CoV NAbs, which were positively correlated. However, it was later shown in a follow-up study that the cross-reactivity between SARS-CoV and MERS-CoV was unlikely to be due to similarity in the RBD as monoclonal antibodies raised to SARS-CoV RBD did not bind MERS-CoV RBD or neutralize it even at high concentrations.^35^ It is unclear from our assay whether the neutralization of MERS-CoV pseudovirus is mediated by S1 or S2 antibodies, which requires further investigation. Overall, the cross-reactivity between SARS-CoV-2 and MERS-CoV could be due to the stimulation of preexisting immunity from past MERS-CoV infection, induced by the persistence of high avidity antibodies after infection.

Since anti-SARS-CoV-2 antibodies with Fc-effector function activity have not been extensively characterized in vivo or in vitro, we assessed ADCC to identify whether exposure to SARS-CoV-2 and MERS-CoV antigens induces Fc-mediated activity (could be related to targeted epitope: S1 vs S2). Our findings did not show any significant increase in ADCC activity following vaccination. Similarly, those vaccinated without previous exposure to MERS-CoV did not elicit a good ADCC response to the S protein. Conversely, SARS-CoV-2 infection induced a significant ADCC response to S and N proteins compared with vaccination. Hence, it would be interesting to assess whether prior MERS-CoV infection induces ADCC against MERS-CoV. In addition, although not investigated here, a previous study described different profiles of Fc-effector functionality within the panel of isolated cross-reactive antibodies from a patient who had recovered from SARS.^36^ In that study, cross-reactive antibodies targeting diverse epitopes on the S protein demonstrated Fc-effector function in vitro, including antibody-dependent cellular phagocytosis, antibody-dependent cellular trogocytosis, and antibody-dependent complement deposition. These data suggest that cross-reactive CoV antibodies could induce Fc-effector function activity that might not involve ADCC, which merits further investigation.

### Limitations

Our study had some important limitations, including the small sample size where we only had 18 samples collected from patients who had MERS, and we only had 4 patients with follow-up samples. Also, we measured cross-reactivity with other CoVs using a multiplex bead-based assay which detects only cross-reactive binding antibodies against the S1 antigen. Hence, measuring cross-reactivity with other antigens (such as S2) and NAbs using a pVNT against these CoVs would provide important insights regarding whether these cross-reactive responses are protective or not. Also, we only examined the effect of COVID-19 mRNA vaccines following priming with MERS-CoV antigens but investigating other COVID-19 vaccines, such adenovirus-vectored vaccines and inactivated vaccines, could shed more light on the effect of prime-bossing strategy with different CoVs. Regardless, to our knowledge, this is the first study to assess the effect of priming with MERS-CoV antigens through infection followed by vaccination with COVID-19 mRNA vaccines. Therefore, our study provides evidence supporting the immune priming-boosting strategies that elicit broadly reactive antibodies to SARS-CoV-2.

## Conclusions

This cohort study identified a set of patients who induced cross-reactive binding and NAb responses after exposure to antigens from 2 major CoVs, MERS-CoV and SARS-CoV-2. We found that vaccination might enhance preexisting immunity against MERS-CoV and SARS-CoV, and to a lesser extent, against other human CoVs. Still, we did not investigate the potential targeted epitopes by these antibodies. Isolation of broadly reactive and possibly NAbs from these patients could help identify cross-reactive epitopes and hence, help in the structure-based design of pan-CoV vaccines and therapeutic molecules.

## Notes

Supplement 1. **eTable.** Demographic and Clinical Characteristics of MERS-CoV Patients **eFigure 1.** Schematic Illustrating the Experimental Study Design and Strategy of Testing **eFigure 2.** Dot Plot of Antibody Responses Against SARS-CoV-2 in Paired Samples Collected from Patients Showing an Immune Response After COVID-19 Vaccination **eFigure 3.** Antibody Responses to the SARS-CoV-2 Nucleocapsid (NP), S Trimer, S1, RBD, and Envelope (E) Proteins in Pre- and Post-Vaccination Samples Compared to SARS-CoV-2 Infected Patients **eFigure 4.** IgG Antibody Subclasses Against SARS-CoV-2 Antigens and Other Human Coronaviruses S1 Protein, Including IgG1 (A), IgG2 (B), IgG3 (C), and IgG4 (D) **eFigure 5.** IgA Antibody Subtypes Against SARS-CoV-2 Antigens and Other Human Coronaviruses S1 Protein, Including IgA1 (A) and IgA2 (B) **eFigure 6.** Cross-Reactive Antibodies Induced Against Other Human Coronaviruses in Previously Infected MERS-CoV Patients With Serial Samples Collected Before and After COVID-19 Vaccination

Supplement 2.
Data Sharing Statement
