# Supplementary material for: Assessment of Broadly Reactive Responses in Patients With MERS-CoV Infection and SARS-CoV-2 Vaccination
Source: JAMA Netw Open. 2023 Jun 30;6(6):e2319222. doi: 10.1001/jamanetworkopen.2023.19222 (PMC10314312; doi:10.1001/jamanetworkopen.2023.19222)
Supplement: Supplement 1. — eTable. Demographic and Clinical Characteristics of MERS-CoV Patients eFigure 1. Schematic Illustrating the Experimental Study Design and Strategy of Testing eFigure 2. Dot Plot of Antibody Responses Against SARS-CoV-2 in Paired Samples Collected from Patients Showing an Immune Response After COVID-19 Vaccination eFigure 3. Antibody Responses to the SARS-CoV-2 Nucleocapsid (NP), S Trimer, S1, RBD, and Envelope (E) Proteins in Pre- and Post-Vaccination Samples Compared to SARS-CoV-2 Infected Patients eFigure 4. IgG Antibody Subclasses Against SARS-CoV-2 Antigens and Other Human Coronaviruses S1 Protein, Including IgG1 (A), IgG2 (B), IgG3 (C), and IgG4 (D) eFigure 5. IgA Antibody Subtypes Against SARS-CoV-2 Antigens and Other Human Coronaviruses S1 Protein, Including IgA1 (A) and IgA2 (B) eFigure 6. Cross-Reactive Antibodies Induced Against Other Human Coronaviruses in Previously Infected MERS-CoV Patients With Serial Samples Collected Before and After COVID-19 Vaccination [file jamanetwopen-e2319222-s001.pdf]

Supplemental Online Content

Zedan HT, Smatti MK, Thomas S, et al. Assessment of broadly reactive responses in patients with MERS-CoV infection and SARS-CoV-2 vaccination. *JAMA Netw Open.* 2023;6(6):e2319222. doi:10.1001/jamanetworkopen.2023.19222

eTable. Demographic and Clinical Characteristics of MERS-CoV Patients

eFigure 1. Schematic Illustrating the Experimental Study Design and Strategy of Testing

eFigure 2. Dot Plot of Antibody Responses Against SARS-CoV-2 in Paired Samples Collected from Patients Showing an Immune Response After COVID-19 Vaccination

eFigure 3. Antibody Responses to the SARS-CoV-2 Nucleocapsid (NP), S Trimer, S1, RBD, and Envelope (E) Proteins in Pre- and Post-Vaccination Samples Compared to SARS-CoV-2 Infected Patients

eFigure 4. IgG Antibody Subclasses Against SARS-CoV-2 Antigens and Other Human Coronaviruses S1 Protein, Including IgG1 (A), IgG2 (B), IgG3 (C), and IgG4 (D)

eFigure 5. IgA Antibody Subtypes Against SARS-CoV-2 Antigens and Other Human Coronaviruses S1 Protein, Including IgA1 (A) and IgA2 (B)

eFigure 6. Cross-Reactive Antibodies Induced Against Other Human Coronaviruses in Previously Infected MERS-CoV Patients With Serial Samples Collected Before and After COVID-19 Vaccination

This supplemental material has been provided by the authors to give readers additional information about their work.

© 2023 Zedan HT et al. *JAMA Network Open.*

eTable. Demographic and Clinical Characteristics of MERS-CoV Patients

Patient ID	Gender	Age (years)	Nationality	Pre-vaccination sample	Post-vaccination sample	Date of second dose administration	Date of sample collection	Days between second dose and collection
MERS-1	M	28	Bangladeshi	Yes	-		29/01/2021	
				-	Yes	6/24/2021	10/21/2021	119
MERS-2	M	34	Bangladeshi	Yes	-		4/12/2021	
				-	Yes	9/3/2021	10/20/2021	47
MERS-3[a]	M	70	Qatari	Yes	-		4/12/2021	
				-	Yes	4/14/2021	10/20/2021	189
MERS-4	M	31	Bangladeshi	Yes	-		4/12/2021	
				-	Yes	9/11/2021	10/20/2021	39
MERS-5	M	34	Qatari	Yes	-		Feb/2016	
MERS-6	M	35	Qatari	Yes	-		2016	
MERS-7	M	51	Syrian	Yes	-		2016	
MERS-8	M	41	Canadian	Yes	-		2016	
MERS-9	M	35	Syrian	Yes	-		2016	
MERS-10	M	51	Syrian	Yes	-		2016	
MERS-11	M	51	Canadian	Yes	-		2016	
MERS-12	M	34	Canadian	Yes	-		2016	
MERS-13	M	66	Qatari	-	Yes	3/2/2021	8/21/2021	172
MERS-14	M	54	Qatari	-	Yes	3/16//2021	10/24/2021	222

[a] Has a history of infection with SARS-CoV-2.

© 2023 Zedan HT et al. *JAMA Network Open.*

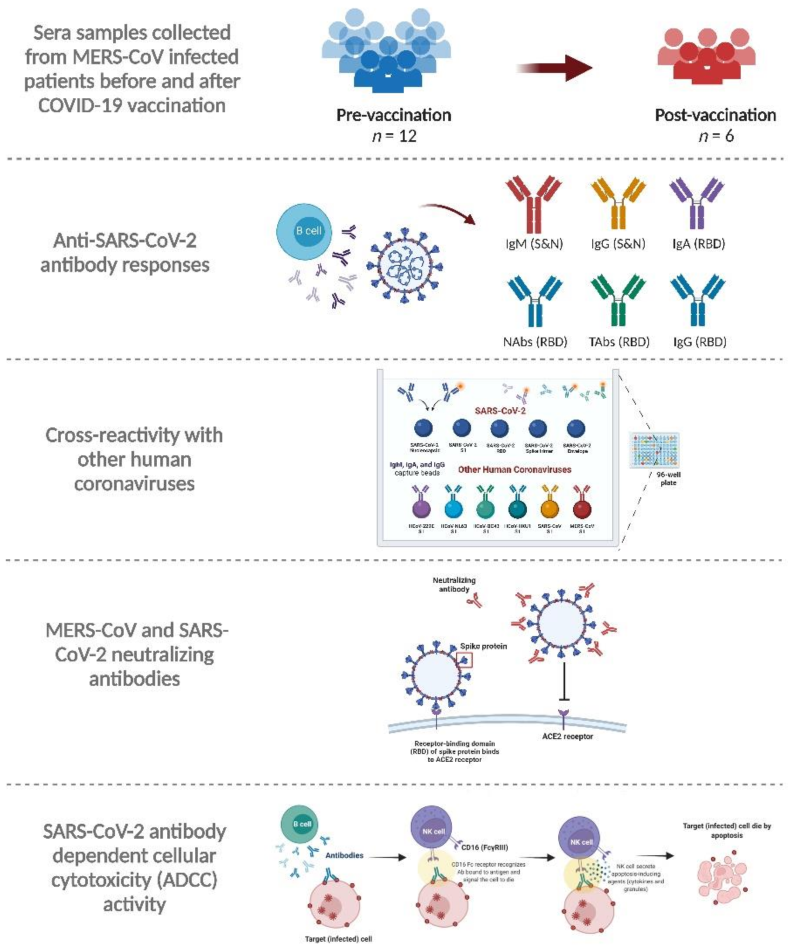

eFigure 1. Schematic illustrating the experimental study design and strategy of testing.

A total of 18 samples were collected from MERS-CoV infected patients before (n=12) and after (n=6) COVID-19 vaccination. All samples were assessed for anti-SARS-CoV-2 antibody responses, followed by an assessment of cross-reactivity against other human coronaviruses. Functional antibodies were also evaluated by pseudotyped-virus neutralization assay for both SARS-CoV-2 and MERS-CoV and by antibody-dependent cellular cytotoxicity (ADCC) activity assay.

© 2023 Zedan HT et al. *JAMA Network Open.*

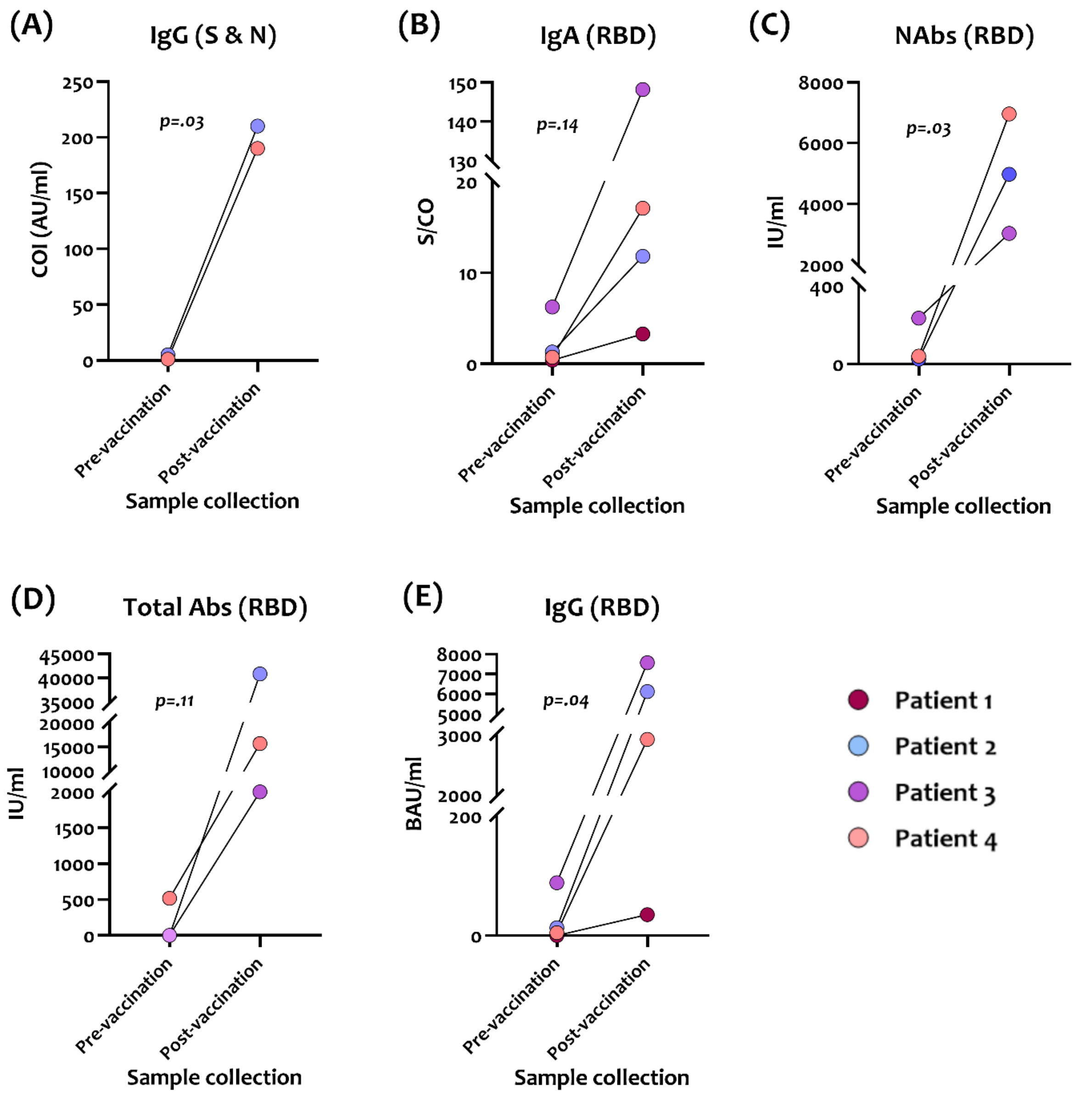

eFigure 2. Dot Plot of Antibody Responses Against SARS-CoV-2 in Paired Samples Collected from Patients Showing an Immune Response After COVID-19 Vaccination

(A) IgG against S and N proteins, **(B)** IgA against the RBD, **(C)** NAbs against the RBD, **(D)** Total antibodies against the RBD, and **(E)** IgG against the RBD. P values were calculated using paired t-test.

© 2023 Zedan HT et al. *JAMA Network Open.*

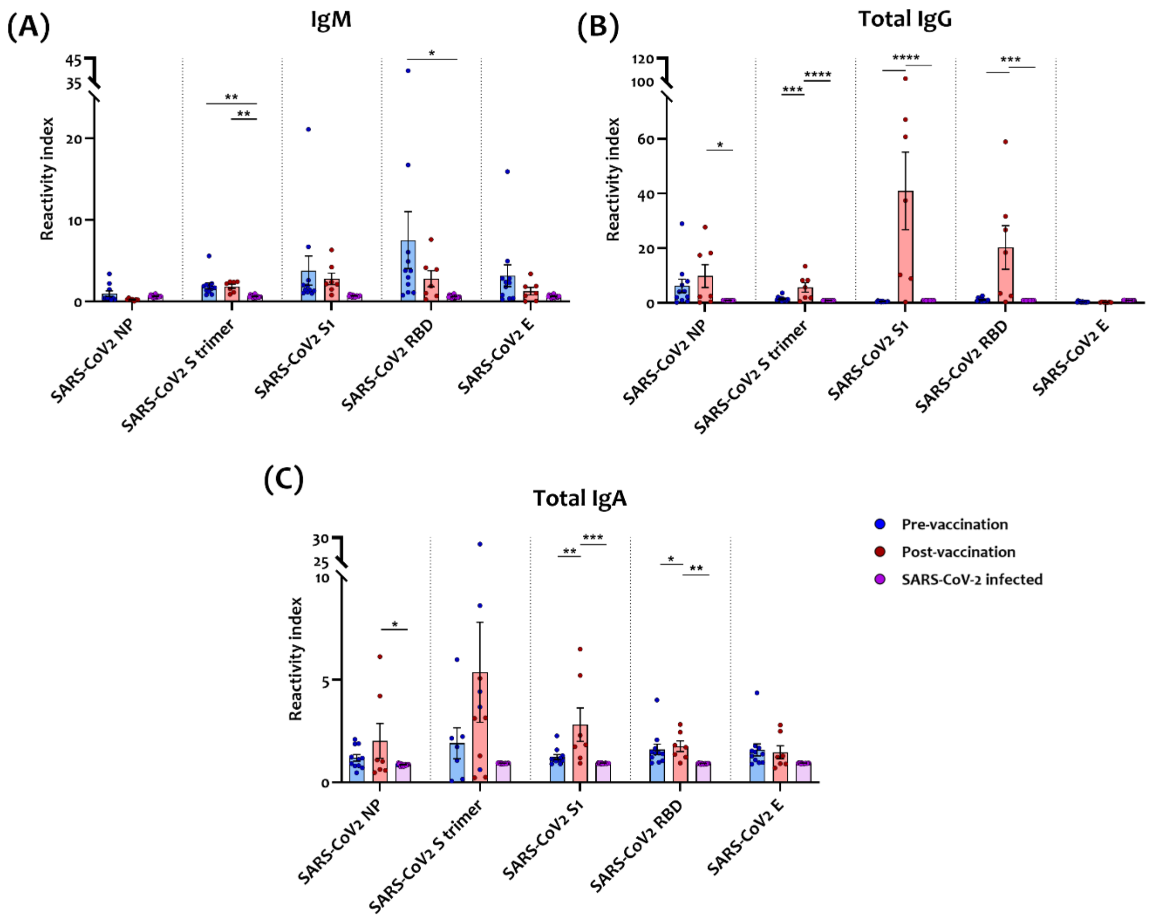

eFigure 3. Antibody Responses to the SARS-CoV-2 Nucleocapsid (NP), S Trimer, S1, RBD, and Envelope (E) Proteins in Pre- and Post-Vaccination Samples Compared to SARS-CoV-2 Infected Patients

(A) IgM antibody responses, **(B)** Total IgG antibody responses, and **(C)** Total IgA antibody responses. P values were calculated using t-test. *$p < 0.05$, **$p < 0.01$. ***$p < 0.001$.

© 2023 Zedan HT et al. *JAMA Network Open.*

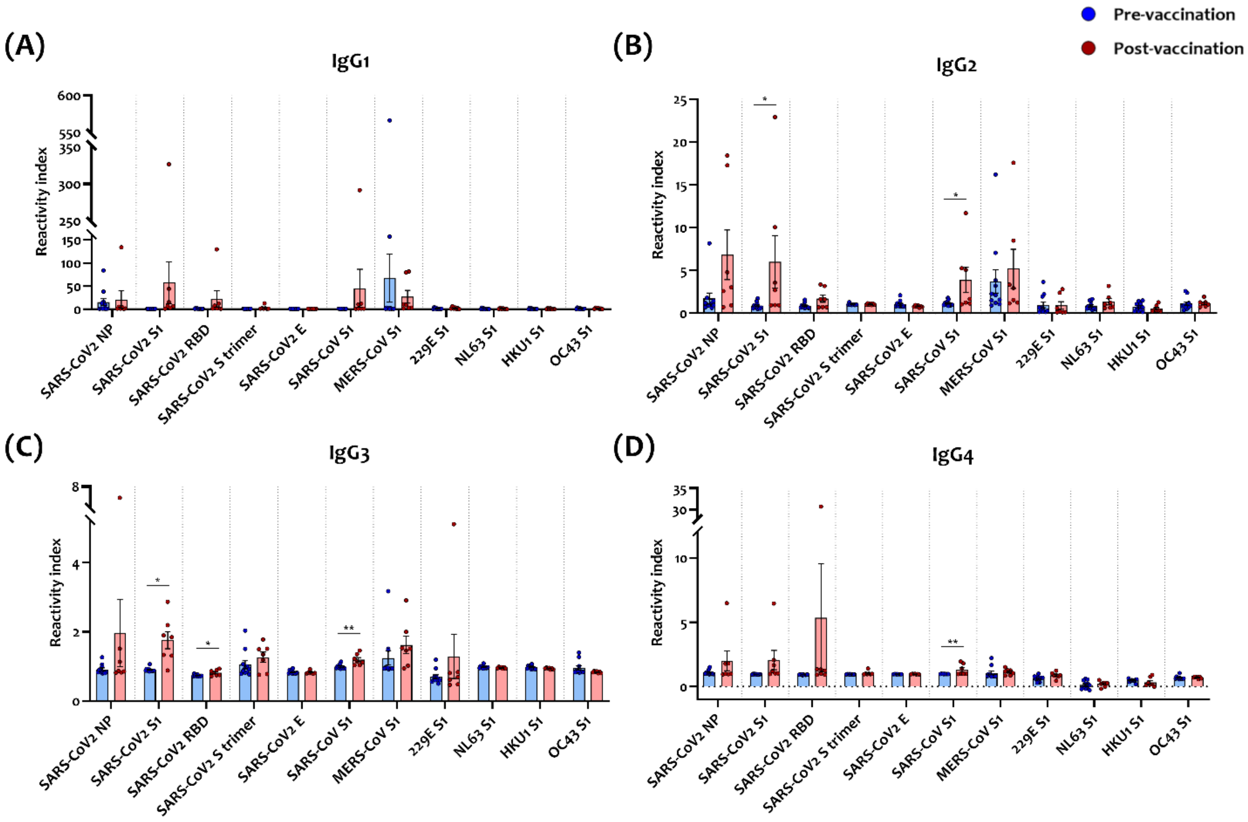

eFigure 4. IgG Antibody Subclasses Against SARS-CoV-2 Antigens and Other Human Coronaviruses S1 Protein, Including IgG1 (A), IgG2 (B), IgG3 (C), and IgG4 (D)

P values were calculated using t-test. *p< 0.05, **p< 0.01.

© 2023 Zedan HT et al. *JAMA Network Open.*

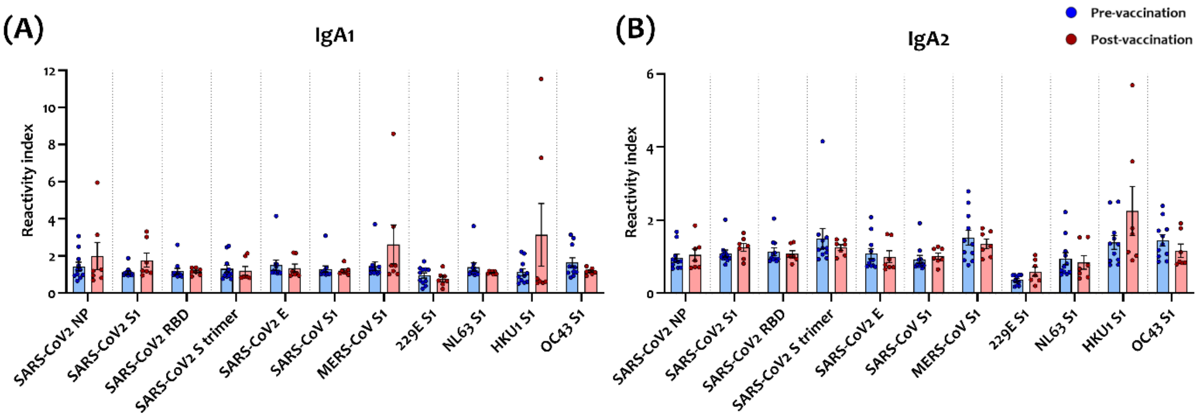

eFigure 5. IgA Antibody Subtypes Against SARS-CoV-2 Antigens and Other Human Coronaviruses S1 Protein, Including IgA1 (A) and IgA2 (B)

P values were calculated using t-test. *$p < 0.05$.

© 2023 Zedan HT et al. *JAMA Network Open.*

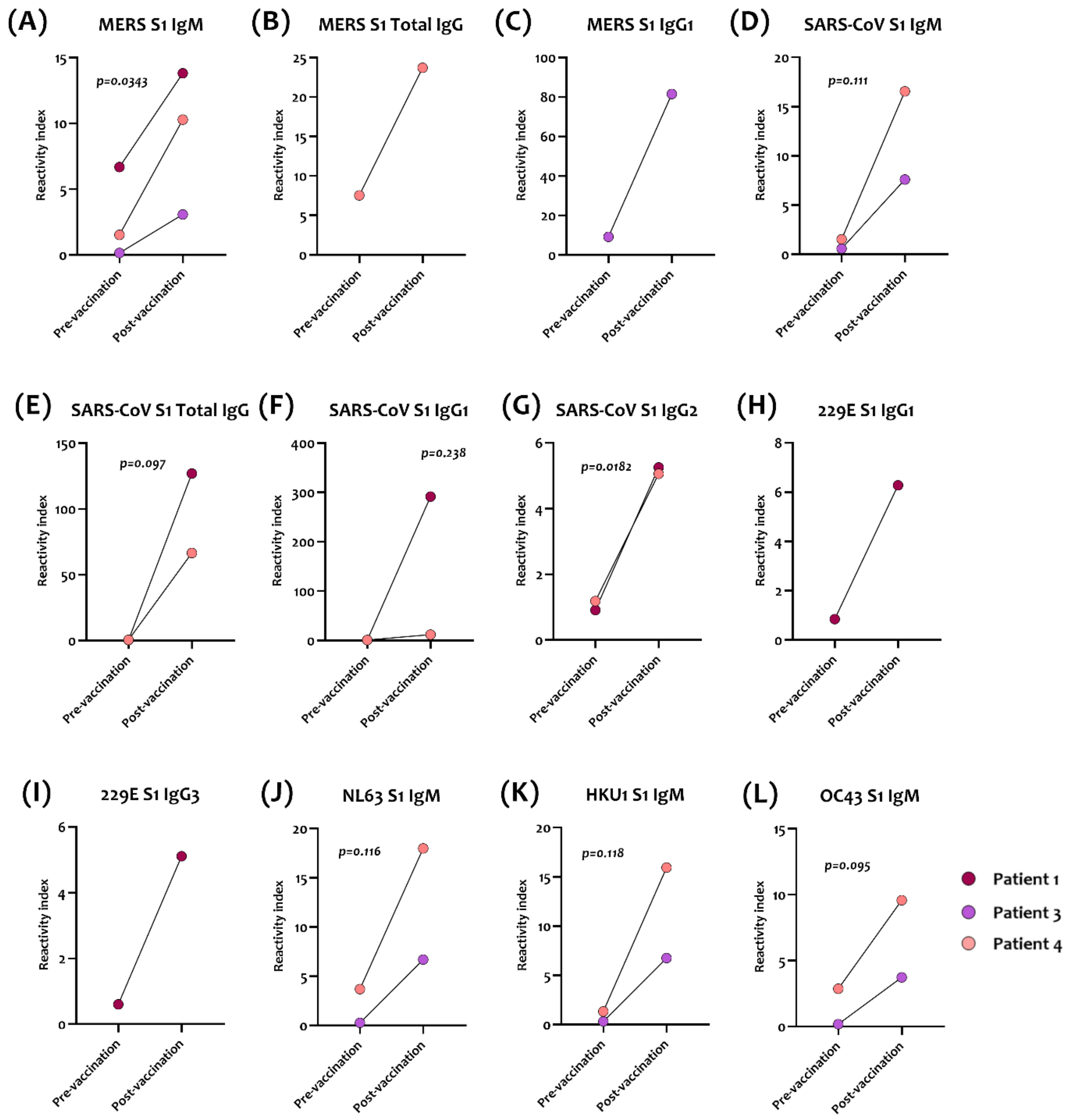

eFigure 6. Cross-Reactive Antibodies Induced Against Other Human Coronaviruses in Previously Infected MERS-CoV Patients With Serial Samples Collected Before and After COVID-19 Vaccination

(A) IgM against MERS-CoV S1, **(B)** Total IgG against MERS-CoV S1, **(C)** IgG1 against MERS-CoV S1, **(D)** IgM against SARS-CoV S1, and **(E)** Total IgG against SARS-CoV S1, **(F)** IgG1 against SARS-CoV S1, **(G)** IgG2 against SARS-CoV S1, **(H)** IgG1 against HCoV-229E S1, **(I)** IgG3 against HCoV-229E S1, **(J)** IgM against HCoV-NL63 S1, **(K)** IgM against HCoV-HKU1 S1, **(L)** IgM against HCoV-OC43 S1. P values were calculated using paired t-test.

© 2023 Zedan HT et al. *JAMA Network Open.*
