# Supplementary material for: Assessment of Broadly Reactive Responses in Patients With MERS-CoV Infection and SARS-CoV-2 Vaccination
Source: JAMA Netw Open. 2023 Jun 30;6(6):e2319222. doi: 10.1001/jamanetworkopen.2023.19222 (PMC10314312; doi:10.1001/jamanetworkopen.2023.19222)
Supplement: Supplement 2. — Data Sharing Statement [file jamanetwopen-e2319222-s002.pdf]

# Data Sharing Statement

Zedan. Assessment of Broadly Reactive Responses in Patients With MERS-CoV Infection and SARS-CoV-2 Vaccination. *JAMA Netw Open*. Published June 30, 2023.
doi:10.1001/jamanetworkopen.2023.19222

**Data**
**Data available:** Yes
**Data types:** Deidentified participant data
**How to access data:** Contact corresponding author for permission hyassine@qu.edu.qa
**When available:** With publication

**Supporting Documents**
**Document types:** None

**Additional Information**
**Who can access the data:** researchers whose proposed use of the data has been approved
**Types of analyses:** Similar analyses to the conducted study
**Mechanisms of data availability:** Contact corresponding author for permission
hyassine@qu.edu.qa
